# Evaluation verhaltenspräventiver Gesundheitsförderungsmaßnahmen in Inklusionsbetrieben

**DOI:** 10.1007/s11553-022-00959-9

**Published:** 2022-06-20

**Authors:** Ilona Efimov, Anika Tell, Ann-Christin Kordsmeyer, Volker Harth, Stefanie Mache

**Affiliations:** Zentralinstitut für Arbeitsmedizin und Maritime Medizin (ZfAM), Universitätsklinikum Hamburg-Eppendorf (UKE), Seewartenstr. 10 | Haus 1, 20459 Hamburg, Deutschland

**Keywords:** Prozessevaluation, Betriebliche Gesundheitsförderung, Leitungskräfte, Menschen mit Behinderung, Seminare, Process evaluation, Workplace health promotion, Supervisors, People with disabilities, Seminars

## Abstract

**Hintergrund:**

Inklusionsbetriebe nach §§ 215–218 SGB IX bieten schwerbehinderten Menschen eine Beschäftigung auf dem allgemeinen Arbeitsmarkt. Seit 2018 sind Inklusionsbetriebe verpflichtet, Maßnahmen der betrieblichen Gesundheitsförderung (BGF) anzubieten.

**Ziel der Arbeit:**

Das Ziel der vorliegenden Studie ist es, verhaltenspräventive BGF-Angebote in Form von Seminaren für schwerbehinderte Beschäftigte und deren Leitungskräfte in Inklusionsbetrieben zu evaluieren.

**Material und Methoden:**

Anhand eines multimethodischen Studiendesigns erfolgte im Zeitraum von Juni bis November 2021 eine Evaluation der 12 Beschäftigtenseminare (3 Seminarkonzepte, z. B. Selbstfürsorge im Arbeitsalltag) mittels Fokusgruppen (*n* = 44) und eine Evaluation der 3 Leitungskräfteseminare zur gesunden Führung mittels eines standardisierten Fragebogens (*n* = 10). Die erhobenen quantitativen Daten wurden deskriptiv ausgewertet und die qualitativen Daten mithilfe der qualitativen Inhaltsanalyse nach Mayring induktiv analysiert.

**Ergebnisse:**

Die Ergebnisse zeigten, dass die durchgeführten Maßnahmen zur BGF von Beschäftigten hinsichtlich der Zufriedenheit, der Seminarlänge, der Verständlichkeit und des erwarteten Nutzens mehrheitlich positiv bewertet wurden. Die Leitungskräfte bewerteten die Seminarinhalte, die Didaktik, die Seminarleitung und den Beitrag der Teilnehmenden mehrheitlich positiv.

**Diskussion:**

Die Studie lieferte erste empirische Erkenntnisse zur Evaluation von BGF-Maßnahmen in Inklusionsbetrieben. Vor allem die auf die Inklusionsbetriebe angepassten Seminarinhalte konnten die Zufriedenheit der Teilnehmenden mit der Gesundheitsförderungsmaßnahme positiv beeinflussen. Insgesamt bedarf es weiterer Studien zur Entwicklung und Evaluation von verhältnis- und verhaltensbezogenen BGF-Maßnahmen in Inklusionsbetrieben.

## Abstract (en)

**Background:**

Social firms according to §§ 215–218 SGB IX offer employment to severely disabled people on the general labour market. Since 2018, social firms are obliged to offer workplace health promotion (WHP) measures.

**Objectives:**

This study aims to evaluate behavioural-related offers implemented as seminars for WHP for severely disabled employees and their supervisors in social firms.

**Materials and methods:**

Using a multimethod study design, from June to November 2021, 12 seminars for employees (3 seminar concepts, e.g. on self-care in everyday work) were evaluated by means of focus groups (*n* = 44) and 3 seminars for supervisors on healthy leadership by means of a standardized questionnaire (*n* = 10). The collected quantitative data were analysed descriptively and the qualitative data were analysed inductively according to Mayring’s qualitative content analysis.

**Results:**

The results indicated that the implemented WHP measures for employees were rated positively by the majority in terms of satisfaction, seminar length, comprehensibility and usefulness. Similarly, supervisors evaluated the seminar content, didactics, instructor as well as the contribution of other participants mostly positively.

**Conclusion:**

The study provided initial empirical findings on the evaluation of WHP measures in social firms. In particular, seminar content adapted to the setting of social firms had a positive influence on participants’ satisfaction with health promotion measures. Overall, further studies are needed to develop and evaluate structural and behavioural-related WHP offers in social firms.

## Einleitung

Fast die Hälfte der über 7,9 Mio. Menschen mit einer Schwerbehinderung in Deutschland (entspricht 9,5 % der Gesamtbevölkerung) sind im arbeitsfähigen Alter (ca. 40 % sind zwischen 25 und 64 Jahre alt; [40]). Davon befinden sich jedoch nur 46,9 % in einem Arbeitsverhältnis im Vergleich zu 75,2 % der Menschen ohne Behinderungen [3]. Erwerbsarbeit kann jedoch positive Einflüsse auf den Genesungsprozess von Menschen mit psychischen Erkrankungen [13, 30, 41] wie auch auf die Entwicklung sozialer Fähigkeiten, eine größere Autonomie, die Stärkung eines Gefühls der Selbstbestimmung sowie die Erlangung einer beruflichen Identität haben [6]. Demnach sollten Beschäftigungsverhältnisse von Menschen mit Behinderungen aus gesellschaftlicher und gesundheitlicher Perspektive gestärkt und gefördert werden. Inklusionsbetriebe stellen eine besondere Unternehmensform dar, welche gemäß Artikel 27 der UN-Behindertenrechtskonvention Menschen mit Behinderungen eine gleichberechtigte Teilhabe am allgemeinen Arbeitsmarkt ermöglichen, indem sie u. a. gesunde und sichere Arbeitsbedingungen sicherstellen und die Möglichkeit bieten, den Lebensunterhalt durch frei gewählte Arbeit zu verdienen [4]. In Deutschland beschäftigen Inklusionsbetriebe nach § 215 SGB IX 30–50 % Menschen mit psychischen oder geistigen, Sinnes‑, Körper- oder Mehrfachbehinderungen, die eine Benachteiligung auf dem allgemeinen Arbeitsmarkt erfahren. Die Mehrheit der schwerbehinderten Beschäftigten in Inklusionsbetrieben weist mit jeweils 27 % eine seelische oder geistige Behinderung auf [2]. Zusätzlich bieten Inklusionsbetriebe nach § 216 SGB IX schwerbehinderten Beschäftigten u. a. eine arbeitsbegleitende Betreuung, bedarfsorientierte Maßnahmen der beruflichen Weiterbildung, die Möglichkeit zur Teilnahme an entsprechenden außerbetrieblichen Maßnahmen an oder passen auch die Arbeitsumgebung an individuelle Bedürfnisse an.

Eine aktuelle systematische Übersichtsarbeit verdeutlicht den Einfluss von Arbeitsbedingungen in Inklusionsbetrieben auf gesundheits- und arbeitsbezogene Outcomes von Menschen mit Behinderungen. Soziale Unterstützung, flexible Arbeitsbedingungen, strukturierte Arbeitsaufgaben, Weiterbildungsmöglichkeiten, Arbeitsplatzsicherheit und Partizipationsmöglichkeiten stellen dabei relevante Ressourcen für Beschäftigte dar [22]. Auf der anderen Seite besteht bislang ein geringer Wissensstand zu den Belastungsfaktoren von Beschäftigten in Inklusionsbetrieben – hier werden v. a. arbeitsorganisatorische Belastungen (z. B. hohe Arbeitsmenge und Zeitdruck) oder soziale Konflikte am Arbeitsplatz berichtet [8, 22]. Ebenso besteht eine unzureichende Studienlage zur Arbeits- und Gesundheitssituation von Leitungskräften in Inklusionsbetrieben. Eine explorative Interviewstudie von Kordsmeyer et al. [19] zeigt, dass Leitungskräfte insbesondere die Sinnhaftigkeit ihrer Arbeit ebenso wie die Kombination von beruflichen und pädagogischen Aufgaben als Ressourcen am Arbeitsplatz wahrnehmen. Arbeitsorganisatorische Belastungsfaktoren wie die Vereinbarkeit sozialer und wirtschaftlicher Ziele oder das Abfangen von Arbeitsausfällen erleben sie hingegen als zentrale Belastungen [19].

Um den Umgang mit spezifischen Belastungsfaktoren am Arbeitsplatz zu unterstützen bzw. Ressourcen zu stärken, können Betriebe im Rahmen der betrieblichen Gesundheitsförderung (BGF) sowohl verhaltenspräventive (Veränderung der individuellen Verhaltensweisen) als auch verhältnispräventive Maßnahmen (Veränderungen der Arbeitsbedingungen) implementieren [9]. Nach dem aktuellen Stand der Forschung haben BGF-Maßnahmen sowohl einen gesundheitlichen wie auch ökonomischen Nutzen [15, 24, 32, 36, 43] und sind insbesondere im Sinne eines ganzheitlichen Ansatzes in der Kombination von sowohl verhaltens- als auch verhältnispräventiven Maßnahmen am wirkungsvollsten [14]. Auch wenn seit dem 01.01.2018 Inklusionsbetriebe in Deutschland nach §§ 215–218 SGB IX verpflichtet sind, über den allgemeinen Arbeits- und Gesundheitsschutz hinaus BGF-Maßnahmen anzubieten, bestehen nach dem derzeitigen Stand der Forschung bislang noch keine wissenschaftlich fundierten Evaluationsstudien von BGF-Maßnahmen in Inklusionsbetrieben [22]. Eine aktuelle Befragung von Inklusionsbetrieben in Deutschland macht auf der einen Seite deutlich, dass mehrheitlich (81,7 %) kein spezifisches Konzept für die BGF besteht und meist auf die bestehenden Angebote der gesetzlichen Krankenversicherung zurückgegriffen wird. Auf der anderen Seite berichten Inklusionsbetriebe, dass die bestehenden Angebote selten an die spezifischen Bedarfe der Inklusionsbetriebe angepasst sind. Es zeigt sich, dass im Rahmen der BGF mehrheitlich (71 %) Maßnahmen zur Reduzierung von Arbeitsbelastungen umgesetzt werden – jedoch nur 45,2 % der Betriebe Angebote im Bereich der individuellen Verhaltensprävention anbieten [39]. Eine explorative qualitative Befragung verweist zudem auf eine Vielzahl potenzieller Herausforderungen in der Implementierung von BGF-Maßnahmen in Inklusionsbetrieben [21]. Im Vergleich zum Setting der Inklusionsbetriebe zeigen einzelne Interventionsstudien in Werkstätten für Menschen mit Behinderungen (WfbM) in Deutschland [1, 23, 26, 28] und Belgien [7] positive Evaluationsergebnisse.

In Anbetracht des gesundheitsförderlichen Potenzials verhaltenspräventiver Angebote ([15]; z. B. in Form von Seminaren [1]) sowie eines hohen Bedarfs an BGF-Maßnahmen seitens der Inklusionsbetriebe [39] wurden in der vorliegenden Studie neuwertige verhaltenspräventive Seminarangebote zur BGF für schwerbehinderte Beschäftigte und Leitungskräfte in Inklusionsbetrieben entwickelt und durchgeführt. Nach dem derzeitigen Stand der Forschung bestehen noch keine wissenschaftlichen Erkenntnisse zur Implementierung von BGF-Maßnahmen im Setting der Inklusionsbetriebe.

Ziel der Evaluation ist daher die Überprüfung der durchgeführten Maßnahmen und die Anpassung an die speziellen Bedürfnisse der Zielgruppe, die Prüfung der Übereinstimmung mit dem Projektplan, die Prüfung der Zielgruppenerreichung sowie die Sicherung und kontinuierliche Verbesserung der Qualität während der Durchführung. Zu diesem Zweck werden die folgenden Evaluationsfragestellungen herausgearbeitet:

- Zufriedenheit:
  - Sind die Beschäftigten und Leitungskräfte mit den Seminarinhalten, der Seminarleitung und den Rahmenbedingungen des Seminars zufrieden?
- Subjektiv erwartete Nutzenbewertung der Maßnahmen:
  - Besteht ein Bezug des Seminars zur Lebenswirklichkeit der Beschäftigten?
  - Bewerten die Beschäftigten die Seminarinhalte als relevant für ihren Berufsalltag?
  - Geben die Leitungskräfte einen zusätzlichen Wissenserwerb an?
- Verständlichkeit der Inhalte:
  - Sind die Seminarinhalte für die Beschäftigten und Leitungskräfte verständlich, nicht überfordernd und interessant aufbereitet?
  - Wird für die Beschäftigten eine ausreichend leicht verständliche Sprache angewendet?
- Verbesserungsbedarf der Seminare:
  - Welchen Verbesserungsbedarf nehmen die Beschäftigten und Leitungskräfte nach der Durchführung des Seminars wahr?

## Methodik

### Studiendesign

Im Rahmen eines 3‑jährigen Forschungsprojekts („Betriebliche Gesundheitsförderung in Inklusionsbetrieben nach §§ 215 ff SGB IX – BeGIn“) wurden auf Basis umfangreicher Analysen der Belastungs- und Ressourcensituation sowie des Unterstützungsbedarfs in den Inklusionsbetrieben verschiedene Seminarkonzepte für die Beschäftigten mit Schwerbehinderungen und deren Leitungskräfte entwickelt [8, 19]. Die durchgeführten Maßnahmen wurden in Form eines Mixed-Methods-Studiendesigns evaluiert, bestehend aus Fokusgruppen zur qualitativen Evaluation der Seminare für die Beschäftigten und einer quantitativen Fragebogenerhebung für die Evaluation der Seminare für die Leitungskräfte. Fokusgruppen wurden als niedrigschwellige Evaluationsmethodik für die Seminare mit den Beschäftigten gewählt, da sie in leicht verständlicher Sprache umsetzbar sind und einen erfahrungsbezogenen Austausch in der Seminargruppe fördern [37]. Die Evaluation erfolgte im Schwerpunkt in Form einer Prozessevaluation (zur Zufriedenheit, Bewertung der Seminarlänge, Verständlichkeit und zum Verbesserungsbedarf), ergänzt um ein Element der Ergebnisevaluation (zur subjektiven Nutzenbewertung). Die Durchführung einer Prozessevaluation ermöglichte eine Untersuchung der Projektumsetzung [10] sowie der Zielgruppenerreichung und Akzeptanz der Maßnahmen [27]. Ein entscheidender Vorteil einer Prozessevaluation bestand dadurch, dass potenzielle Herausforderungen rechtzeitig erkannt werden und die erzielten Ergebnisse unmittelbar in die Verbesserung der Maßnahmen einfließen konnten [27].

### Konzeptdarstellung verhaltenspräventiver Gesundheitsförderungsmaßnahmen

Auf Basis einer vorherigen Analysephase der Belastungs- und Ressourcensituation von Beschäftigten mit Behinderungen aus Inklusionsbetrieben [8, 19] wurden 3 Seminarkonzepte für die Beschäftigten mit Schwerbehinderungen neu entwickelt, welche die folgenden komplementären Themenbereiche abdecken: 1) *Selbstfürsorge im Arbeitsalltag*, 2) *Kommunikation und Konfliktlösung* und 3) *Gesunde Teamarbeit*. Zwei qualifizierte Fachkräfte des externen Projektpartners (Gesundheitswissenschaften und Sozialpädagogik) führten die Seminare im Zeitraum von Juni bis November 2021 durch. Die Forschungsgruppe evaluierte die durchgeführten Seminare. Die Teilnahme an den Seminaren war für alle 8 teilnehmenden Inklusionsbetriebe kostenfrei. Alle Seminare wurden als Ganztagesseminare konzipiert (ca. 09:30–15:30 Uhr) und erfolgten während der Arbeitszeit. Bei Bedarf wurden die Seminarlänge und Pauseneinteilung entsprechend der Bedürfnisse der jeweiligen Zielgruppe angepasst. Im Zuge der COVID-19-Pandemie wurde unter Berücksichtigung der vulnerablen Teilnehmendengruppe ein Konzept mit hohen Anforderungen an die Einhaltung von Hygiene- und Abstandsregelungen umgesetzt.

Das Seminarkonzept *Selbstfürsorge im Arbeitsalltag* wurde als Ausflug in einen Park geplant und zielte auf die Stärkung von Stressbewältigungsstrategien der teilnehmenden Beschäftigten ab. Dabei wurden sowohl theoretische Inhalte und Informationen, z. B. zum Ablauf von Stressreaktionen im Körper in leicht verständlicher Sprache vermittelt als auch Gruppenübungen und verschiedene Stressbewältigungsmethoden (z. B. Achtsamkeitsübungen nach Jon Kabat-Zinn [17]) praktisch erprobt. Das zweite Seminarkonzept *Kommunikation und Konfliktlösung* zielte auf eine Förderung der Wahrnehmung eigener Bedürfnisse, eine Verbesserung der Kommunikation und des Umgangs mit Konflikten, die Förderung von Kompetenzen im Umgang mit unterschiedlichen Bedürfnissen sowie eine Stärkung von Kommunikations- und Konfliktbewältigungsstrategien der Beschäftigten ab. Neben theoretischen Inhalten in leicht verständlicher Sprache, z. B. zur Vermittlung des Kommunikationsquadrats nach Schulz von Thun [38], wurden dabei auch Paar- oder Gruppenübungen und praktische Übungen zur Übertragung des Wissens in den Alltag eingebaut. Das dritte Seminarkonzept *Gesunde Teamarbeit* verfolgte das Ziel, eine gesunde Teamarbeit zu fördern und zu stärken sowie als langfristige Ressource zur Förderung der Gesundheit von Beschäftigten verfügbar zu machen. Dabei wurde zum einen an der Identifikation eigener Werte und der Teamwerte gearbeitet, die Diversität als Stärke des Teams erarbeitet und die Grundlagen gesunder Teamarbeit thematisiert (z. B. Elemente des personenzentrierten Ansatzes nach Carl R. Rogers [35]). An insgesamt 12 Terminen wurden 3 Seminare *Selbstfürsorge im Arbeitsalltag*, 5 Seminare *Kommunikation und Konfliktlösung* und 4 Seminare *Gesunde Teamarbeit* durchgeführt. Pro Seminar nahmen bis zu 4 Beschäftigte teil. Bei Bedarf konnten Beschäftigte auch an einem weiteren Seminar teilnehmen. Ein geplantes Seminar ausschließlich für gehörlose Beschäftigte konnte mehrmalig aufgrund krankheitsbedingter Abwesenheiten nicht durchgeführt werden.

Für die Leitungskräfte wurde das Seminarkonzept *Gesunde Führung* neu entwickelt. In Anlehnung an das Modell der Gesundheitsorientierten Führung nach Franke und Felfe [11] fokussierte die erste Hälfte des Seminars die Selbstführung vor dem Hintergrund der Vereinbarkeit von wirtschaftlichen und sozialen Faktoren und die zweite Hälfte die Mitarbeiterführung mit Übungen zu den Themen Kommunikation, Wertschätzung und Anerkennung (z. B. Kommunikationsquadrat nach Schulz von Thun [38]). Das Seminar zielte auf eine Sensibilisierung für die eigenen Grenzen, die Reflexion und Förderung adäquater Abgrenzungsstrategien ab. Weiterhin wurde ebenso die Kompetenz im Umgang mit der eigenen Geduld gestärkt. Die Seminare für Leitungskräfte wurden an insgesamt 3 Terminen mit jeweils bis zu 4 Leitungskräften durchgeführt (s. Tab. 1). Übergeordnet wurde im Bereich der kommunikationsbezogenen Seminarthemen ein stufenweises Vorgehen gewählt, um ähnliche thematische Schwerpunkte und dazu passende Übungen für Beschäftigte und Leitungskräfte auf einem unterschiedlichen Anforderungsniveau anbieten zu können.

**Tab. 1:** Übersicht der Anzahl der Seminare (*n* = 15) und der Teilnehmenden (*n* = 54)

Seminarkonzept	Anzahl Seminare	Anzahl Teilnehmende	
	*n*	*n*	%
*Seminare für Beschäftigte*	*12*	*44*	*81,5*
Selbstfürsorge im Arbeitsalltag	3	13	24,1
Kommunikation und Konfliktlösung	5	16	29,6
Gesunde Teamarbeit	4	15	27,8
*Seminare für Leitungskräfte*	*3*	*10*	*18,5*
Gesunde Führung	3	10	18,5

Die Mehrheit der Seminarkonzepte wurde in gemischten Gruppen mit Personen aus unterschiedlichen Inklusionsbetrieben durchgeführt. Diese Vorgehensweise zielte darauf ab, einen Perspektivwechsel, betriebsübergreifenden Austausch und Anonymität für die Teilnehmenden zu ermöglichen. Lediglich das Seminar *Gesunde Teamarbeit* wurde in bestehenden Arbeitsteams durchgeführt, um den Teilnehmenden eine Erarbeitung der Teamwerte zu ermöglichen. Alle Seminarkonzepte wurden in deutscher Sprache in den Räumlichkeiten des Forschungsinstituts (mit Ausnahme eines Seminars bei dem externen Projektpartner) für die Seminardurchführung umgesetzt. Lediglich das Seminarkonzept *Selbstfürsorge im Arbeitsalltag* wurde als Ausflug in einen Park mit anschließender Reflexion im Seminarraum durchgeführt.

### Rekrutierung der Stichprobe

Die Rekrutierung der Stichprobe erfolgte in Kooperation mit 8 teilnehmenden Inklusionsbetrieben aus Norddeutschland. Die Geschäftsführungen der jeweiligen Inklusionsbetriebe wurden im ersten Rekrutierungsschritt telefonisch und per E‑Mail über die geplante Seminardurchführung informiert und zur kostenfreien Teilnahme eingeladen. Im nächsten Schritt informierte wenn gewünscht eine Mitarbeiterin des Forschungsprojekts unter Zuhilfenahme von Flyern die Beschäftigten und Leitungskräfte an ihrem Arbeitsplatz über das Seminarangebot. Zu den Einschlusskriterien für die Teilnahme an den Seminaren zählten: 1) eine Beschäftigung gemäß §§ 215–218 SGB IX* oder* Beschäftigung als Leitungskraft einer Arbeitsgruppe in einem Inklusionsbetrieb, 2) ein Alter von mindestens 18 Jahren und 3) Kenntnisse der deutschen Sprache.

### Datenerhebung

Die Datenerhebung zur Evaluation erfolgte für die Beschäftigten und Leitungskräfte auf zwei unterschiedliche Weisen. Zum einen führten jeweils 2 Mitarbeiterinnen des Forschungsprojekts (A.-C.K., I.E., A.T.) qualitative Fokusgruppen im Anschluss an die Beschäftigtenseminare durch. Die Datenerhebung erfolgte mithilfe eines halbstrukturierten Interviewleitfadens, welcher die folgenden 5 Evaluationsfragen beinhaltete: 1) *Hat Ihnen das Seminar gefallen?* 2) *War die Länge des Seminars in Ordnung?* 3) *Haben Sie alles verstanden?* 4) *Können Sie das Gelernte auf der Arbeit nutzen?* und 5) *Haben Sie noch Verbesserungsvorschläge?*. Die Evaluationsfragen zur Zufriedenheit, Seminarlänge, Verständlichkeit und subjektiven Nutzenbewertung wurden zusammen mit jeweils einer 5‑stufigen Smiley-Bewertungsskala (1 = *„Stimme gar nicht zu“* bis 5 = *„Stimme vollkommen zu“,* vgl. Jäger [16]) auf einer Flipchart visualisiert, um ergänzende quantitative Evaluationsdaten zu erheben. Für die Beantwortung der Fragen wurden die Teilnehmenden gebeten, mithilfe von Klebepunkten ihre persönliche Einschätzung auf der Skala abzubilden und diese mündlich mit Beispielen zu begründen. Lediglich die Frage zum Verbesserungsbedarf wurde qualitativ erhoben. Die Dauer der Fokusgruppen betrug durchschnittlich 09:10 min (Range: 04:31–15:19 min).

Zum anderen wurde eine quantitative, papiergestützte Fragebogenerhebung unmittelbar im Anschluss an die Leitungskräfteseminare durchgeführt. Der eingesetzte standardisierte Fragebogen basiert auf der revidierten Version des *Münsteraner Fragebogens zur Evaluation von Seminaren (MFE-Sr)* nach Thielsch und Hirschfeld [42] unter Nutzung folgender 3 Skalen: 6 Items zu „Dozent und Didaktik“ (Nr. 8–13 im MFE-Sr, α = 0,89, Beispielitem: „Der/Die Lehrende hat das Thema interessant aufgearbeitet.“), 3 Items zur „Überforderung“ (Nr. 14–16 im MFE-Sr, α = 0,77, Beispielitem: „Die Inhalte des Seminars waren zu schwierig für mich.“), 3 Items zu den „Teilnehmer:innen“ (Nr. 17–19 im MFE-Sr, α = 0,82, Beispielitem: „Die meisten Teilnehmer brachten sich aktiv ein.“). Für diese 3 Skalen wurde ein 7‑stufiges Antwortformat (1 = *„Stimme gar nicht zu“* bis 7 = *„Stimme vollkommen zu“*) angewendet. Insgesamt wiesen die eingesetzten Skalen eine ausreichende bis gute interne Konsistenz auf. Zusätzlich wurden folgende, nicht validierte Items aus dem MFE-Sr in den Fragebogen integriert: 3 Items zu den Rahmenbedingungen (räumliche Ausstattung, Lautstärke und eigene Zeitplanung: Nr. 5–7 im MFE-Sr; 7‑stufiges Antwortformat: 1 = *„Stimme gar nicht zu“* bis 7 = *„Stimme vollkommen zu“*), ein Item zum Wissenserwerb (Nr. 25 im MFE-Sr; dichotomes Antwortformat: 0 = *„Nein“*, 1 = *„Ja“*), ein Item zur Weiterempfehlung des Seminars (Nr. 26 im MFE-Sr; dichotomes Antwortformat: 0 = *„Nein“*, 1 = *„Ja“*), ein Item zur allgemeinen Bewertung des Seminars nach Schulnoten (Nr. 27 im MFE-Sr; 5‑stufiges Antwortformat: 1 = *„sehr gut“* bis 5 = *„sehr schlecht“*) sowie ein Item für weitere Anmerkungen zum Seminar (Nr. 28 im MFE-Sr; offenes Textfeld). Die Items wurden jeweils an den Seminarkontext angepasst („Teilnehmende“ statt „Studierende“, 5‑stufiges anstelle eines 15-stufigen Punktesystems). Ergänzend wurden demografische Daten zum Alter, Geschlecht, Branche, Beschäftigungsdauer sowie zur Anzahl unterstellter Beschäftigter abgefragt. Vor der Evaluation der Seminare wurden das Ziel, der Ablauf und der geltende Datenschutzstandard des Forschungsprojekts und der Evaluation erläutert. Für die Teilnehmenden der Beschäftigtenseminare wurden die Selbstauskunft und Informationen zum Datenschutz jeweils in leicht verständlicher Sprache aufbereitet.

### Datenanalyse

Die durchgeführten Fokusgruppen wurden mit einem Audioaufnahmegerät dokumentiert und anschließend nach Kuckartz [25] transkribiert und anonymisiert. Die Analyse der Transkripte erfolgte induktiv nach der qualitativen Inhaltsanalyse nach Mayring [29]. Diese Analysemethode wurde gewählt, da sie einen systematischen, regelbasierten Ansatz verfolgt und sich dabei auf den semantischen Datengehalt konzentriert [29]. Die Datentranskription und -analyse erfolgte durch eine Gesundheitswissenschaftlerin (A.T.) ohne Einbeziehung der Teilnehmenden und wurde durch 2 wissenschaftliche Mitarbeiterinnen (I.E., A.-C.K.) geprüft. Dabei wurde die Analysesoftware MAXQDA (Version: 12.3.9, VERBI GmbH, Berlin, Deutschland) verwendet. Aufgekommene Unstimmigkeiten hinsichtlich der Kodierung bzw. der inhaltlichen Auswertung wurden im Team gelöst und besprochen bis ein Konsens erreicht wurde. Für eine bessere Illustration der qualitativen Ergebnisse der Beschäftigtenseminare wurden exemplarisch einzelne Zitate aus den Fokusgruppen dargestellt. Zusätzlich zur qualitativen Datenanalyse erfolgte eine deskriptive Auswertung der Ergebnisse anhand einer 5‑stufigen Smiley-Bewertungsskala (vgl. Jäger [16]). Ebenso wurden die quantitativen Daten der Fragebogenerhebung deskriptiv ausgewertet (I.E.). Hierfür wurde die Statistiksoftware IBM SPSS Statistics (Version: 26.0, IBM Corporation, Armonk, NY, USA) verwendet.

### Einhaltung ethischer Richtlinien

Die Fokusgruppen und Fragebogenerhebungen wurden mit Zustimmung der zuständigen Ethikkommission des Universitätsklinikums Hamburg-Eppendorf (LPEK-0051), sowie gemäß der Deklaration von Helsinki von 1975 (in der aktuellen, überarbeiteten Fassung) durchgeführt. Von allen beteiligten Teilnehmenden liegt eine schriftliche Einverständniserklärung vor.

## Ergebnisse

### Charakteristika der Stichprobe

An den 3 Seminaren für Beschäftigte nahmen insgesamt 44 Personen an 12 Terminen teil (Tab. 1). Die meisten Beschäftigten waren weiblich (59,1 %) und zwischen 40 und 49 Jahren alt (38,6 %). Die Mehrheit der Beschäftigten arbeitete seit mehr als 3 Jahren in dem Inklusionsbetrieb (61,4 %; Tab. 2). An dem Seminar für Leitungskräfte nahmen insgesamt 10 Personen an 3 Terminen teil (Tab. 1). Die Leitungskräfte waren zu gleichen Anteilen männlich und weiblich sowie mehrheitlich zwischen 50 und 59 Jahren alt (40 %). Die meisten Leitungskräfte waren seit über 3 Jahren in dem Betrieb angestellt (60 %) und leiteten bis zu 5 Beschäftigte (40 %; Tab. 2). Die kooperierenden 8 Inklusionsbetriebe waren dabei in unterschiedlichen Branchen verortet: Gastronomie, Hotellerie, Reinigung, Veranstaltungswirtschaft, Infrastrukturdienstleistung, IT-Beratung und Zweiradindustrie.

**Tab. 2:** Charakteristika der Stichprobe (*N* = 54)

Charakteristika	Beschäftigte (*n* = 44)		Leitungskräfte (*n* = 10)	
	*n*	%	*n*	%
*Geschlecht*				
Männlich	18	40,9	5	50,0
Weiblich	26	59,1	5	50,0
*Alter (Jahre)*				
18–29	6	13,6	1	10,0
30–39	11	25,0	2	20,0
40–49	17	38,6	3	30,0
50–59	6	13,6	4	40,0
60 +	4	9,1	–	–
*Beschäftigungsdauer*				
< 1 Jahr	4	9,1	1	10,0
1–3 Jahr	11	25,0	3	30,0
> 3 Jahr	27	61,4	6	60,0
*Anzahl unterstellte Beschäftigte*				
< 5 Personen	–	–	4	40,0
6–10 Personen	–	–	2	20,0
> 10 Personen	–	–	2	20,0
k. A.	–	–	2	20,0

### Evaluation der Seminarkonzepte für Beschäftigte

Die Auswertung der Fokusgruppen mit den Beschäftigten beinhaltete zwei Komponenten: Zum einen wurden die qualitativen Ergebnisse in insgesamt 5 Hauptkategorien sowie 16 Unterkategorien induktiv herausgearbeitet (Tab. 4). Zum anderen wurden die 5‑stufigen Smiley-Bewertungsskalen (vgl. Jäger [16]) deskriptiv ausgewertet (Tab. 3).

**Tab. 3:** Mittelwerte (*M*) und Standardabweichungen (*SD*) der Seminarevaluation von Beschäftigten mit Behinderungen

Evaluationskriterien	Selbstfürsorge im Arbeitsalltag		Kommunikation und Konfliktlösung		Gesunde Teamarbeit	
	*M*	*SD*	*M*	*SD*	*M*	*SD*
Zufriedenheit	4,46	0,78	4,75	0,45	4,80	0,41
Nutzen	4,69	0,63	4,56	0,63	4,53	0,74
Seminarlänge	4,38	1,12	4,50	0,82	4,33	0,82
Verständlichkeit	4,77	0,60	4,63	0,62	4,87	0,35

*N* = 44 Beschäftigte aus 6 Inklusionsbetrieben. Verwendung einer 5‑stufigen Smiley-Bewertungsskala: 5 = *Stimme vollkommen zu*; 1 = *Stimme gar nicht zu*

**Tab. 4:** Bewertung der Seminare für Beschäftigte mit Behinderungen anhand 5 Hauptkategorien und 16 Unterkategorien

Hauptkategorien	Unterkategorien
Zufriedenheit	Seminarinhalte (+)
	Gruppengröße und -zusammensetzung (+/−)
	Seminarort (+)
	Austausch und Kennenlernen der Kolleg:innen (+)
Seminarlänge	Eignung der Seminarinhalte für das Zeitformat (+/−)
	Pausengestaltung, aktivierende Elemente (+/−)
Verständlichkeit	Vermittlung & Aufbereitung der Seminarinhalte (+/−)
	Rücksicht auf individuelle Bedürfnisse & Voraussetzungen (+)
	Ungewohnte, zu umfangreiche Thematik (−)
Verbesserungsbedarf	Weitere Themenvorschläge, Ideen für Seminarinhalte (−)
	Anwendung leichterer Sprache (−)
	Verwendung von Namensschildern (−)
Subjektive Nutzenbewertung	Erkenntnisgewinn (+)
	Integrierung der praktischen Übungen in den Arbeitsalltag (+)
	Verbesserung der Zusammenarbeit (+)
	Abhängigkeit von individuellen Voraussetzungen und externen Faktoren (−)

(+) = positive Kritik, (−) negative Kritik bzw. Verbesserungsbedarf

### Zufriedenheit

Die quantitative Auswertung der Bewertungen zur Zufriedenheit mit den Seminarinhalten anhand der 5‑stufigen Smiley-Bewertungsskala ergab, dass die Beschäftigten mit allen 3 Seminarkonzepten im Durchschnitt zufrieden bis sehr zufrieden waren (Tab. 3). Am zufriedensten waren die Teilnehmenden mit dem Seminarkonzept „Gesunde Teamarbeit“ (*M* = 4,80; *SD* = 0,41). Die qualitative Auswertung der Fokusgruppen verdeutlichte, dass die hohe Zufriedenheit mit den Seminarinhalten, der Gruppengröße und -zusammensetzung, dem Seminarort, Austausch und Kennenlernen der Kolleg:innen begründet wurde.

### Seminarinhalte

Die Beschäftigten äußerten sich insgesamt positiv zu den Inhalten der Seminare. Dabei wurden die verschiedenen Seminarkonzepte mehrheitlich als interessant, vielfältig und relevant beschrieben. Vereinzelt nannten die Teilnehmenden auch konkrete Bestandteile der Seminare, die ihnen besonders gut gefielen, wie z. B. die Einheiten zur „Identifizierung gemeinsamer Teamwerte“ oder zur „Wahrnehmung von Gefühlen am Arbeitsplatz“ im Selbstfürsorgeseminar.

*So, also mir hat es sehr gut gefallen, was zum Schluss gekommen ist, also mit den Empfindungen am Arbeitsplatz.* (#B3, männlich, 30–39 Jahre; Selbstfürsorgeseminar)

Weiterhin wurden die Seminare als praxisnah beschrieben. Im Falle der Selbstfürsorgeseminare bewerteten die Beschäftigten die praktischen Übungen und Einheiten, wie die Atem- und Entspannungsübungen, den Spaziergang durch den Park sowie die (spielerischen) Bewegungselemente mehrheitlich positiv.

*Ja, die Atemübungen und (…) auch irgendwie (…) ja, da gab es irgendwie so eine Übung, wo wir angeleitet wurden, was wir mit unseren Gedanken machen sollen […] und ja, das fand ich spannend.* (#B4, männlich, 18–29 Jahre; Selbstfürsorgeseminar)

Weiterhin gaben mehrere Teilnehmende an, dass ihnen einige der Seminarinhalte bereits bekannt waren, was aber aufgrund der Auffrischungswirkung als positiv empfunden wurde.

*Ja, also viele Themen sind mir bekannt […]. Ich habe einiges schon wieder vergessen und das war eine perfekte Auffrischung für mich.* (#B6, männlich, > 60 Jahre; Kommunikationsseminar)

Übergeordnet berichteten mehrere Beschäftigte, die Seminare an Kolleg:innen und Leitungskräfte weiterempfehlen zu können.

### Gruppengröße und -zusammensetzung

In den Team- oder Kommunikationsseminaren äußerten sich die Beschäftigten heterogen hinsichtlich der Gruppengröße und -zusammensetzung. Während einige der Beschäftigten eine kleine Gruppengröße bevorzugten bzw. nicht nachteilig sahen, präferierten andere Beschäftigte größere Gruppen. Dabei wurde jedoch angemerkt, dass die Eignung der Gruppengröße situativ von der jeweiligen Aufgabe bzw. Anforderung abhing So eigneten sich kleinere Gruppen beispielsweise besser für vertrauliche Gespräche, während die Beschäftigten annahmen, dass größere Gruppen vielfältigere Kommunikationsproblematiken aufzeigen könnten und gemeinsame Lösungsansätze entwickelt werden könnten.

*Also um ein vertrauensvolles Gespräch zu haben (…) ist das (…) (B15: Zweier-Gruppe sehr gut.) Ist sehr gut, ja. (B15: Wiederum, um einige Sachen in dem Ding zu machen, ist eine größere Gruppe natürlich besser.)* (#B16, männlich, 40–49 Jahre; #B15, männlich, > 60 Jahre; Kommunikationsseminar)

Ein Beschäftigter sah in einer großen Gruppengröße jedoch den Nachteil, dass die thematisierten Problematiken zu unterschiedlich sein könnten und so zu einer Spaltung der Gruppe führen könnten. Ein weiterer Nachteil wurde darin gesehen, dass in größeren Gruppen eher die Gefahr bestünde, die Seminardauer zu überschreiten.

*Man kennt sich, ja, nicht weil man sich kennt, das ist auch interessant neue Leute kennenzulernen, aber (…) im Endeffekt nun mal jeder Betrieb hat seine eigenen Probleme und das spaltet dann wahrscheinlich zu sehr.* (#B15, männlich, > 60 Jahre; Kommunikationsseminar)

### Seminarort

Die Durchführung des Selbstfürsorgeseminars im Park bewerteten die Teilnehmenden positiv. Die Beschäftigten gaben dabei mehrheitlich an, dass die Übungen durch die Sinneseindrücke in ihrer Wirkung verstärkt wurden und sie sich in der Natur entspannt, fröhlich und wohl fühlten.

*Ich fand es jetzt auch schön, dass wir draußen sein konnten. Das hat in dem Rahmen durch die, also Thema Achtsamkeit, sich selbst spüren, irgendwie hat das in der Natur, war das schön. Und ja, konnte ich mich sehr wohl fühlen.* (#B4, männlich, 18–29 Jahre; Selbstfürsorgeseminar)

Auch wenn die Festlegung des Seminarorts von der Wetterlage abhängig gemacht werden musste, wurden Kälte oder gelegentlicher Regen als nicht störend empfunden. Weitere Vorteile der Seminardurchführung im Park im Vergleich zum Seminarraum wurden in der besseren Luftqualität, höheren Bewegungsfreiheit und der Tatsache, dass keine Masken getragen werden mussten, gesehen.

*Ich glaube, das ist aber auch (…) wenn wir die ganzen Stunden in einem Raum gearbeitet hätten, das wäre auch von der Luft her, von der Bewegungsfreiheit her, von der frischen Luft, das wäre nicht dasselbe gewesen.* (#B3, männlich, 30–39 Jahre; Selbstfürsorgeseminar)

### Austausch und Kennenlernen der Kolleg:innen

Über alle 3 Seminarkonzepte hinweg wurde der Austausch in der Seminargruppe positiv bewertet. Insbesondere in den Teamseminaren beschrieben die Teilnehmenden, dass sie die Kolleg:innen durch die Seminare besser kennenlernen konnten und ihnen das gemeinsame Erarbeiten der Seminarinhalte im Team gut gefiel. So konnten sie sowohl die Wahrnehmung eigener Fähigkeiten und Stärken verbessern als auch Einblicke in die Gedanken, Stärken und Fähigkeiten der Kolleg:innen gewinnen. Eine Teilnehmende hob vor allem den Austausch über gemeinsame Werte, das gemeinsame Erarbeiten und die Reflexion der Stärken im Team positiv hervor.

*Ja, ich fand es halt schön, weil wir mal den Fokus auf unser Team gesetzt haben und auch mal sehr inhaltlich wurden bewusst, was uns im Team und in der Arbeit wichtig ist.* (#B9, weiblich, 40–49 Jahre; Teamseminar)

### Seminarlänge

Die quantitativ erhobene Bewertung der Seminarlänge anhand der 5‑stufigen Smiley-Bewertungsskala ergab eine im Durchschnitt positive Bewertung der Seminarlänge durch die Teilnehmenden (Tab. 3). Die qualitative Auswertung der Fokusgruppen bestätigte, dass die Mehrheit zufrieden mit der Seminarlänge und Pausengestaltung war. Mehrere Beschäftigte äußerten jedoch den Wunsch, das Seminar zu verkürzen (um 30 min bis zu 2 h), einzelne Beschäftigte hingegen wünschten sich eine längere Seminardauer. Insbesondere das lange Sitzen sowie Konzentrationseinbußen wurden als negative Folgen einer zu langen Seminardauer beschrieben.

*Für mich ist das, das lange Sitzen habe ich immer Probleme. […] Aber das ist ja allgemein ein Problem, weil mir geht das immer so auf die Knie.* (#B13, weiblich, 50–59 Jahre; Kommunikationsseminar)

### Eignung der Seminarinhalte für das Zeitformat

Einzelne Beschäftigte wünschten sich eine stärkere thematische Abwechslung oder aktive Pausen. Wiederum andere Beschäftigte gaben an, dass die Seminarinhalte für das angegebene Zeitformat geeignet und nicht überfordernd waren.

*„Eine Idee ja (…) ich weiß nicht, ob man das also an einem Tag dann umsetzen kann. Das ist ja (…) das ist immer sehr schwer, aber was weiß ich, dass man doch irgendwie […] komplett was anderes macht. […] Wüsste ich jetzt nicht, in welche Richtung, aber jetzt dieses Thema erstmal total auslässt und dann ja (…). Das ist, ist lang, ist wirklich lange. Ist auch lang und trocken.“* (#B10, männlich, > 60 Jahre, Teamseminar)

*„Also ich habe mich jetzt nicht überfrachtet gefühlt.“* (#B12, männlich, 30–39 Jahre; Kommunikationsseminar)

### Pausengestaltung, aktivierende Elemente

Die Pausengestaltung bewerteten die Beschäftigten mehrheitlich sehr positiv. Dabei wurden sowohl die Anzahl als auch die Dauer der Pausen als gut gestaltet wahrgenommen. Insbesondere die Flexibilität der Seminarleitung bei der Absprache, die Möglichkeit, Pausen auch draußen sowie gemeinsam verbringen zu können, wurde positiv hervorgehoben. Zudem bewerteten die Teilnehmenden häufige aktivierende Elemente im Seminarablauf, wie Bewegungspausen, Auflockerungsübungen oder der Spaziergang durch den Park positiv.

*„Ne, so zwischendurch ein paar Pausen finde ich ganz gut. Wenn sie auch nicht so lange sind. Und die Mittagspause, bisschen Gemeinschaft haben, das fand ich auch sehr schön.“* (#B13, weiblich, 50–59 Jahre; Kommunikationsseminar)

*„Also wir hatten ja so ein paar Bewegungselemente hier mit eingebracht. Das hat schon mal ganz gut geklappt, das hat schon mal ein bisschen aufgelockert.“* (#B11, weiblich, 30–39 Jahre; Teamseminar)

Zudem äußerten einzelne Beschäftigte den Wunsch, weitere Pausen einzuplanen und die Kommunikation hinsichtlich der Gestaltung der Pausen zu optimieren.

### Verständlichkeit

Im Durchschnitt gaben die Beschäftigten im Rahmen der quantitativen Erhebung anhand der 5‑stufigen Smiley-Bewertungsskala an, dass die Seminarinhalte gut verstanden wurden (Tab. 3). Ähnlich wie beim Evaluationskriterium „Zufriedenheit“ bewerteten die Beschäftigten das Seminarkonzept „Gesunde Teamarbeit“ am besten hinsichtlich der Verständlichkeit (*M* = 4,87; *SD* = 0,35). Übergeordnet ergab die qualitative Auswertung der Fokusgruppen, dass die Vermittlung und Aufbereitung der Seminarinhalte ebenso wie die Rücksichtnahme der Seminarleitung auf individuelle Bedürfnisse und Voraussetzungen positiv beschrieben wurden. Hingegen wurde die Verständlichkeit durch ungewohnte, zu umfangreiche Seminarinhalte vereinzelt auch negativer bewertet.

### Vermittlung und Aufbereitung der Seminarinhalte

Die Beschäftigten gaben mehrheitlich an, dass die Seminarinhalte gut durch die Seminarleitung erklärt und schlüssig zusammengefasst wurden. Weiterhin sorgte der Austausch, das Einbringen von Beispielen oder die Möglichkeit Fragen zu stellen für eine hohe Verständlichkeit der Inhalte. Einige Teilnehmende äußerten weiterhin, dass die praktische Orientierung des Seminars, die einfachen Übungen und die zusätzliche Unterstützung der Inhalte durch Illustrationen hilfreich waren. Als ein negativer Aspekt wurde hinzugefügt, dass das im Seminar verwendete Kommunikationsmodell zu Verständnisproblemen führen könnte.

*„Ja, da gab es auch dieses […] Kommunikationsmodell, […] da kann es glaube ich auch schon mal passieren, dass man das einmal nicht alles versteht, aber eins zu eins war es alles wirklich sehr nahe.“* (#B4, männlich, 18–29 Jahre; Selbstfürsorgeseminar)

### Rücksicht auf individuelle Bedürfnisse und Voraussetzungen

Des Weiteren erlebten die Teilnehmenden die Rücksichtnahme der Seminarleitung auf die individuellen Bedürfnisse und Voraussetzungen der Beschäftigten positiv. So gab eine schwerhörige Teilnehmende an, dass sie üblicherweise Verständnisprobleme bei Fortbildungen erlebte, die Seminarleitung jedoch verstanden wurde und sie gut folgen konnte.

*„Normalerweise habe ich immer bei (…) Fortbildungen Probleme gehabt, immer. Aber heute war das so (…) hat Spaß gemacht, ich konnte ihn sehr gut verstehen. Er war (…) er weiß, dass ich schwerhörig bin. Er hat mir gut, deutlich gesprochen und so.“* (#B15, weiblich, 40–49 Jahre; Teamseminar)

Weiterhin fügten 2 Beschäftigte mit Migrationshintergrund hinzu, dass die ergänzende Mitschrift der Seminarinhalte durch die Seminarleitung als unterstützend für das Verständnis der Seminarinhalte erlebt wurde.

*„Ja, mein Deutsch ist (…). So, wenn er so geredet hat, dann habe ich gedacht: ‚Oh, oh, was war das?‘ und so und wenn er (…) wo er das geschrieben hat und so, ein, zwei Sätze, dann hat das geklappt. ‚Ah, das meinte er‘ dann.“* (#B13, weiblich, > 60 Jahre; Teamseminar)

### Ungewohnte, zu umfangreiche Thematik

Hinsichtlich der Verständlichkeit der Thematik merkten einige Teilnehmende negativ an, dass die Inhalte zu umfangreich waren bzw. die Dauer der Beschäftigung mit der Thematik nicht ausreichend war. Dadurch wurden Schwierigkeiten bei der Umsetzung des Gelernten gesehen. Weiterhin wurde geäußert, dass es aufgrund fehlender Gewöhnung an die Thematik schwierig war dem Seminar zu folgen.

*„Aber es ist schwierig (alle lachen), solchen Dingen zu folgen, wenn man normalerweise während der Arbeit gewohnt ist den Kopf abzuschalten.“* (#B12, männlich, 50–59 Jahre; Teamseminar)

### Verbesserungsbedarf

Des Weiteren trugen die Beschäftigten in den Fokusgruppen ergänzende Verbesserungsideen zu den Seminarinhalten, der Anwendung leichterer Sprache und der Verwendung von Namensschildern zusammen.

### Weitere Themenvorschläge, Ideen für Seminarinhalte

Unter anderem wurden Vorschläge zur Integrierung zusätzlicher Themen oder Seminarinhalte in die bestehenden Seminarkonzepte genannt. So äußerten Beschäftigte z. B. den Wunsch, das Thema Konfliktmanagement im Betrieb detaillierter zu behandeln, weitere Kommunikationsmodelle zu integrieren oder mehr über den Ablauf und die Entstehung von Stressreaktionen im Körper zu erfahren.

*„Also das war interessant, nur (…) ich hätte mir gewünscht, wenn auch nochmal Kommunikation vom anderen (…) von der anderen Seite (…) angesprochen wird. Und nicht nur von Schulz von Thun.“* (#B10, männlich, 40–49 Jahre; Kommunikationsseminar)

Dabei wurde auch angemerkt, dass die Integration weiterer Seminarinhalte den zeitlichen Rahmen der Seminare ausreizen könnte und ggf. weitere Seminarkonzepte entwickelt werden sollten. Weiterhin wurde der Wunsch geäußert, mehr Zeit für den Austausch über den Arbeitsalltag einzuplanen, da sich einige der Kolleg:innen nicht gut kannten bzw. der Kontakt durch die COVID-19-Pandemie erschwert war.

*„Ähm, da weiß ich nicht, ob das da unter Umständen vielleicht hilfreich sein kann, wenn wir mehr aus unserem gemeinsamen Arbeitsalltag halt mehr erzählen könnten, irgendwelche Beispiele (…).“* (#B5, männlich, 50–59 Jahre; Teamseminar)

Als ein weiterer Vorschlag zur Verbesserung des Lerneffekts wurde die Verwendung von Videos mit nachgestellten Szenen aus dem Alltag genannt, mit denen richtige und falsche Kommunikationsweisen noch besser vermittelt werden könnten. Abschließend wünschten sich einige Teilnehmende der Team- und Kommunikationsseminare, dass die Seminarkonzepte auch für die Leitungskräfte angeboten werden bzw. diese in die Seminare einbezogen werden sollten.

*„Nicht nur Kollegen, auch […] Vorgesetzte ja, Führungskräfte, die sollten da auch mal hingehen. Es ist nicht kompliziert, aber das ist eine gute Basis, die jeder Mensch irgendwie (…) darauf aufbauen kann.“* (#B6, männlich, > 60 Jahre, Kommunikationsseminar)

### Anwendung leichterer Sprache

Vereinzelt wurde der Wunsch nach der Anwendung einer noch leichteren Sprache in den Seminaren geäußert.

*„Ja, ich hätte eine (…) nicht eine Bitte, sondern also (…) ja, eine Bitte vielleicht. Vielleicht richtig so (…) in leichter Sprache so?“* (#B2, weiblich, 30–39 Jahre; Kommunikationsseminar)

### Verwendung von Namensschildern

Zudem wurde eine Verwendung von Namensschildern in größeren Seminargruppen angeregt.

*„Aber beim letzten Mal hatte ich ein bisschen Schwierigkeiten mit den Namen und da habe ich gedacht so ne, so ein Kreppband für die Namen.“* (#B13, weiblich, 50–59 Jahre; Kommunikationsseminar)

### Subjektive Nutzenbewertung

Die quantitative Auswertung der Bewertungen zum subjektiven Nutzen der Seminarinhalte mithilfe der 5‑stufigen Smiley-Bewertungsskala ergab, dass die Beschäftigten erwarteten, die neuen Lerninhalte über alle 3 Seminarkonzepte hinweg auch für ihre Arbeit nutzen zu können (Tab. 3). Die qualitative Auswertung der Fokusgruppen zeigte, dass der nachhaltige Nutzen der Seminare übergeordnet mit dem Erkenntnisgewinn, der Eignung der praktischen Übungen für die Integration in den Arbeitsalltag und einer verbesserten Zusammenarbeit im Team beschrieben wurde, jedoch unter Beachtung der Abhängigkeit von individuellen Voraussetzungen und externen Faktoren.

### Erkenntnisgewinn

Die Beschäftigten gaben mehrheitlich an, dass sie neues Wissen und Erkenntnisse durch die Seminare gewinnen konnten, insbesondere hinsichtlich der Individualität von Bedürfnissen und Sichtweisen, der Konfliktlösung, der Bedeutung der Kommunikation von Befindlichkeiten im Arbeitsalltag sowie dem Umgang mit Stress und Belastungen. Weiterhin wurde die nachhaltige Vermittlung und Relevanz der Seminarinhalte positiv hervorgehoben.

*„Und (…) ja, da war er irgendwie wirklich nochmal dran dann zu sagen: ‚Okay was tun wir jetzt auch wirklich?‘ (…) und das hat mich dann auch nochmal überzeugt.“* (#B3, männlich, 18–29 Jahre; Kommunikationsseminar)

*„Ich bin dann hingegangen, ich wusste nicht, was mich erwartet (…). Habe gedacht, das geht halt um Einatmen, Ausatmen, aber das war halt viel mehr (…) und dann doch ziemlich wichtig für mich.“* (#B3, männlich, 30–39 Jahre; Selbstfürsorgeseminar)

### Integrierung der praktischen Übungen in den Arbeitsalltag

Hinsichtlich der Anwendbarkeit der erlernten praktischen Übungen im Arbeitsalltag gaben die Teilnehmenden mehrheitlich an, dass die Übungen gut umsetzbar waren, wobei insbesondere die gemeinsam erarbeiteten Listen und Arbeitsblätter, Tipps zum Umformulieren von Sätzen für die Kommunikation mit Kolleg:innen sowie weitere Materialien, die zur Unterstützung auch am Arbeitsplatz aufgehängt werden konnten, als hilfreich wahrgenommen wurden. Auch die Atem- und Entspannungsübungen im Selbstfürsorgeseminar wurden mehrheitlich als gut in den Arbeitsalltag integrierbar angesehen, da sie nicht anstrengend oder aufwändig in der Durchführung und sehr wirksam waren. Weiterhin wurde von einer Teilnehmerin beschrieben, dass sie sich vorstellen konnte, sich nach dem Seminar mutiger zu fühlen und Probleme anzusprechen.

*„Auch mehr Mut zu haben (…) was zu sagen oder auch Dinge zu sehen.“* (#B13, weiblich, 50–59 Jahre; Kommunikationsseminar)

Einige wenige Beschäftigte äußerten jedoch auch Bedenken, welche sich allerdings konkret auf den Nutzen einzelner Übungen bezogen. Zudem wünschte sich ein Teilnehmer ausführlichere, praktische Tipps zur Konfliktdeeskalation.

*„Ja, das Seminar (…) hieß halt so. Deswegen, man hatte […] also hat eine gewisse Erwartung einfach […] und deswegen dachte ich (…) weiß ich nicht (…) irgendwie auch so eine (…) wie man irgendwie auch gut deeskalieren kann, auch vielleicht irgendwie (…) wenn sich zwei in den Haaren haben. Wie man da irgendwie auch (…), wie man da selber aktiv werden kann und halt nicht nur am Ball steht und ein bisschen hilflos ist.“* (#B3, männlich, 18–29 Jahre; Kommunikationsseminar)

### Verbesserung der Zusammenarbeit

Ein weiterer Nutzen der Team- und Kommunikationsseminare wurde in der Verbesserung der Zusammenarbeit im Team gesehen, da die Teilnehmenden die Kolleg:innen und ihre Bedürfnisse und Werte durch die Seminare besser kennenlernen und Möglichkeiten der Konfliktlösung erlernen konnten. Als Resultat der verbesserten Zusammenarbeit wurde ein stressfreieres und strukturierteres Arbeiten gesehen.

*„Dass sowas dann halt auch die Arbeit stressfreier macht. […] Weil man dann halt orientierter ist, mehr weiß, dass man Struktur hat und die regelmäßig übt durch sowas.“* (#B5, männlich, 50–59 Jahre; Teamseminar)

*„Und genau, dass man gemerkt hat eben, dass man gemeinsame Werte hat und ähm (…), dass das denke ich ein gutes Team wird.“* (#B7, weiblich, 30–39 Jahre; Teamseminar)

### Abhängigkeit von individuellen Voraussetzungen und externen Faktoren

Den Nutzen der Seminarinhalte beschrieben die Beschäftigten jedoch als abhängig von individuellen Voraussetzungen, anderen Personen sowie der Arbeitstätigkeit. Verschiedene Fähigkeiten und Strategien zur Anwendung des Erlernten sowie die Fähigkeit, eigene Überforderung zu erkennen, wurden als individuelle Voraussetzungen genannt.

*„Ja, (…) bei mir ist es so (…) auch so ähnlich. Nur mit dem Unterschied, dass ich das Zeitfenster für mich erkennen muss, wenn es zu viel wird. (…) Da steigt man ja nicht sofort hinter.“* (#B3, männlich, 30–39 Jahre; Selbstfürsorgeseminar)

Weiterhin äußerten mehrere Beschäftigte, dass der praktische Nutzen der Seminarinhalte auch von den Reaktionen der Kolleg:innen im Arbeitsalltag und ihrer Motivation, das Gelernte anzuwenden, abhing. Ebenso wurde ein mangelnder Umsetzungswille der Leitungskräfte als eine zusätzliche Erschwernis wahrgenommen.

*„Ich würde oder wir würden uns wünschen, dass es vielleicht so wie ‚Ja, wir tun was für die Mitarbeiter‘, dass das nicht nur gesagt wird, sondern dass es auch, dass es auch umgesetzt wird und auch gelebt wird und nicht so, nur so getan wird.“* (#B8, männlich, 50–59 Jahre; Teamseminar)

Weitere Schwierigkeiten sahen die Teilnehmenden in Bezug zu ihren jeweiligen Arbeitstätigkeiten, da einige praktische Übungen nicht im Arbeitsalltag umsetzbar waren und Pausen dafür teilweise nicht genommen werden konnten.

*„Eine gemeinsame Pause, die würde uns helfen, das umzusetzen. […] So und das ist aber aufgrund unserer Tätigkeit, ist das leider nicht möglich, aber wir hatten auch in der Pause schon darüber gesprochen, dass das dem Team gut tun würde.“* (#B8, männlich, 50–59 Jahre; Teamseminar)

Tab. 4 stellt die qualitativen Evaluationsergebnisse der Fokusgruppen mit Beschäftigte dar.

### Evaluation des Seminarkonzepts für Leitungskräfte

Insgesamt bewerteten alle Leitungskräfte das Seminarkonzept *Gesunde Führung* mit der Schulnote „gut“ bis „sehr gut“ (*M* = 1,70; *SD* = 0,54) und alle bestätigten, dass sie das besuchte Seminar auch anderen Leitungskräften weiterempfehlen würden (*n* = 10). Insbesondere die Seminarleitung und die angewandte Didaktik wurden positiv bewertet (*M* = 6,52; *SD* = 0,65). Ebenso wurden die Inhalte, das Tempo des Seminars und der damit verbundene Zeitaufwand als nicht überfordernd erlebt (*M* = 1,37; *SD* = 0,62). So bestätigte die Mehrheit der Teilnehmenden, dass sie in dem Seminar einen guten Überblick über das Themengebiet erhielten, sie das Thema interessant fanden, die Gliederung nachvollziehen konnten und die Seminarleitung auf Fragen und Anregungen angemessen einging und oft Beispiele zum besseren Verständnis der Lehrinhalte aufführte. Auch die qualitative Auswertung der offenen Anmerkungen zum Seminar ergab, dass insbesondere die praktischen Übungen, die anschaulichen Beispiele, die auf die Inklusionsbetriebe angepassten Themen und vermittelten Strategien ebenso wie die Einbindung der Teilnehmenden und der Erfahrungsaustausch positiv hervorgehoben wurden. Auch die empathische, offene Kommunikation des Seminarleiters und der Aufbau des Seminars wurden positiv bewertet. Insgesamt gaben > 90 % der Teilnehmenden an, in der Veranstaltung viel gelernt zu haben (*n* = 9). Eine Leitungskraft erläuterte in diesem Zusammenhang, dass bereits Seminarinhalte bekannt waren, jedoch eine Auffrischung des Wissensstandes als hilfreich empfunden wurde. Des Weiteren wurde auch der Beitrag der anderen Leitungskräfte im Seminar positiv bewertet (*M* = 6,40; *SD* = 0,77). Die Rahmenbedingungen hinsichtlich der räumlichen Ausstattung (*M* = 5,90; *SD* = 1,12), der Lautstärke (*M* = 5,70; *SD* = 1,77) und der eigenen Zeitplanung (*M* = 6,20; *SD* = 0,79) wurden überwiegend positiv bewertet. 2 Leitungskräfte gaben hierbei an, dass die Lautstärke nicht ausreichend war, um alles gut verstehen zu können. In der qualitativen Auswertung der offenen Anmerkungen zum Seminar wurde von einzelnen Leitungskräften kritisiert, dass die Zeitplanung für den Umfang der Seminarinhalte zu kurz bemessen war. Weiterhin wurde der Wunsch geäußert, die Themen weitreichender zu behandeln. Tab. 5 zeigt die quantitativen Evaluationsergebnisse der Seminare für Leitungskräfte.

**Tab. 5:** Mittelwerte (*M*) und Standardabweichungen (*SD*) der Seminarevaluation von Leitungskräften

Evaluationskriterien	Gesunde Führung	
	*M*	*SD*
Dozent und Didaktik	6,52	0,65
Überforderung	1,37	0,62
Teilnehmende	6,40	0,77

*N* = 10 Leitungskräfte aus 6 Inklusionsbetrieben. 7 = Stimme vollkommen zu; 1 = Stimme gar nicht zu

## Diskussion

Die vorliegende Studie zur Evaluation von verhaltensorientierten BGF-Maßnahmen ist eine der ersten Evaluationsstudien im deutschen Raum im Setting der Inklusionsbetriebe. Die Ergebnisse aus den Fokusgruppen zeigten, dass die überwiegende Mehrheit der Beschäftigten mit Schwerbehinderung die entwickelten Seminarkonzepte hinsichtlich der Kriterien Zufriedenheit, Bewertung der Seminarlänge, Verständlichkeit sowie erwarteten Nutzen positiv bewerteten. Die Teilnehmenden waren insbesondere mit den Seminarinhalten sowie der Möglichkeit, sich mit Kolleg:innen auszutauschen, zufrieden, sahen einen überwiegend großen Nutzen in den Seminarinhalten und gaben mehrheitlich an, dass die Inhalte verständlich aufbereitet und vermittelt wurden. Lediglich die Gruppengröße und -zusammensetzung, die Seminarlänge und Pausengestaltung wurden heterogener bewertet. Des Weiteren wurden übergeordnete konstruktive Verbesserungsbedarfe und -ideen von den Beschäftigten zusammengetragen. Die Ergebnisse der Fragebogenerhebung verdeutlichten, dass auch die Leitungskräfte die Seminare positiv bewerteten – insbesondere die Seminarinhalte, die Didaktik und Seminarleitung sowie der Beitrag der weiteren Teilnehmenden wurden dabei positiv wahrgenommen. Es konnte keine Überforderung festgestellt werden, lediglich der zeitliche und inhaltliche Umfang des Seminars wurden heterogener bewertet. Insgesamt wurden die durchgeführten Seminarkonzepte von den meisten Teilnehmenden weiterempfohlen.

Nach dem aktuellem Stand der Forschung bestehen derzeit zwar keine vergleichbaren Evaluations- oder Interventionsstudien zu BGF-Maßnahmen in Inklusionsbetrieben [22], jedoch zeigt eine aktuelle explorative Studie, dass bereits eine Vielzahl an BGF-Maßnahmen in deutschen Inklusionsbetrieben umgesetzt werden, wie z. B. Sport‑, Ernährungs- und Entspannungsangebote, aber auch Maßnahmen zur Raucherentwöhnung, Kooperationen mit Krankenkassen sowie Aus- und Weiterbildungsangebote [21]. In einem ähnlichen Setting weisen bisherige vereinzelte Interventionsstudien in WfbM in Deutschland [1, 23, 26, 28] und Belgien [7] auf positive Evaluationsergebnisse im Hinblick auf den Gesundheitszustand und -kompetenz von Beschäftigten mit Behinderungen hin. Für Beschäftigte mit Lernschwierigkeiten oder psychischen Erkrankungen zeigt sich beispielsweise, dass die Teilnahme an längerfristigen Gesundheitskursen dabei unterstützt, ein differenzierteres und umfassenderes Verständnis von Gesundheit zu erwerben, die Kommunikationsfähigkeiten und Sozialkompetenz zu verbessern [1], Depressivität und Ängste zu verringern sowie die psychische Beanspruchung während der Arbeit zu verbessern [26]. Zwei weitere Studien mit geistig behinderten Beschäftigten in WfbM machen deutlich, dass Herz-Kreislauf-Trainings und ein Rückenschulkonzept das erhöhte kardiometabolische Risiko der Gruppe signifikant senken kann [23] und großes Interesse und eine hohe Teilnahmemotivation an dem gesundheitspräventiven Angebot hervorrufen kann [28]. Langzeitauswertungen einer belgischen Studie mit Menschen mit Behinderungen konnten zeigen, dass auch kurze Interventionen gut angenommen werden, positive Auswirkungen auf die wahrgenommene Lebensqualität haben, jedoch auch Zusammenhänge zu unbegründeter Besorgnis unter den Teilnehmenden bestehen [7]. Aktuelle Ergebnisse einer Querschnittsstudie in Wohneinrichtungen und WfbM verdeutlichen zudem, dass 38,9 % der befragten Menschen mit Behinderungen ein niedriges Gesundheitsbewusstsein sowie über 64,0 % Schwierigkeiten im Umgang mit gesundheitsbezogenen Informationen aufweisen [34]. Insgesamt sind die bisherigen Studienergebnisse aus WfbM aufgrund der Anforderungen auf dem allgemeinen Arbeitsmarkt und der Unterschiede in der Rechts- und Beschäftigungsform nur bedingt mit der Arbeitssituation in Inklusionsbetrieben (§§ 215–218 SGB IX) vergleichbar. Auch weitere beispielhafte Studien aus dem allgemeinen Arbeitsmarkt verdeutlichen, dass Interventionsstudien zur Stressreduktion im betrieblichen Kontext wirksam sind und einen positiven Einfluss auf das psychische Wohlbefinden von Beschäftigten haben [33].

Praktische Herausforderungen in der Implementierung von BGF-Maßnahmen spiegelten sich in der vorliegenden Studie in der Rekrutierung der Stichprobe, wie z. B. aufgrund eines Personalmangels durch Krankheitsausfälle, Urlaubszeiten, einer pandemiebedingten Umstrukturierung der arbeitsorganisatorischen Prozesse oder eines teilweise geringen Teilnahmeinteresses. Es zeigte sich, dass Geschäftsführungen eine zentrale Rolle bei der Unterstützung des Forschungsvorhabens und der Umsetzung der Seminare einnahmen. Die Evaluationsergebnisse zur subjektiven Nutzenbewertung der Seminare verdeutlichten zudem, dass die Umsetzung der gelernten Seminarinhalte am Arbeitsplatz in Abhängigkeit von individuellen Voraussetzungen, anderen Personen sowie der Arbeitstätigkeit von den Beschäftigten eingeschätzt wurde. Demnach sollten verhaltens- und verhältnispräventive Angebote bei der Implementierung von BGF-Maßnahmen in Inklusionsbetrieben kombiniert werden [14]. Ebenso verweist eine qualitative Befragung von Experten und Führungskräften aus deutschen Inklusionsbetrieben auf eine Vielzahl potenzieller Herausforderungen bei der Implementierung von BGF-Maßnahmen in Inklusionsbetrieben: eine geringe Inanspruchnahme und ein mangelndes Interesse von Seiten der Beschäftigten, eine unzureichende Anpassung der Angebote an die spezifischen Bedürfnisse und Kapazitäten der Beschäftigten (z. B. Anwendung leicht verständlicher Sprache), fehlende Ressourcen und Strukturen in der BGF, aber auch Herausforderungen in Bezug auf die Vereinbarkeit von BGF-Maßnahmen mit der Arbeitszeit und Organisation, hinsichtlich der Verfügbarkeit von BGF-Maßnahmen sowie in Bezug auf die zentrale Rolle der Geschäftsleitung und der Vorgesetzten [21]. Obwohl Menschen mit Behinderungen einen höheren Vorsorge- bzw. Interventionsbedarf haben [5], kann ein Präventionsdilemma im Hinblick auf niedrige Teilnahmequoten an gesundheitsfördernden und -präventiven Maßnahmen diskutiert werden. Präventionsangebote werden demnach von Personen mit niedrigeren Bedarfen und somit höheren Gesundheitschancen stärker nachgefragt und in Anspruch genommen [12]. Dieser Umstand spiegelt sich auch in den Ergebnissen einer aktuellen Befragung im Auftrag des Bundesministeriums für Arbeit und Soziales wieder: Der Bedarf an Gesundheitsförderung von Beschäftigten in Inklusionsbetrieben ist hoch, dabei berichten einige Inklusionsbetriebe, dass die derzeit bestehenden BGF-Maßnahmen teilweise unzureichend an die Bedürfnisse von Menschen mit Behinderungen angepasst sind [39]. Des Weiteren ließ sich in der vorliegenden Studie ein potenzieller Einfluss der Pandemiesituation auf die Evaluationsergebnisse trotz Einhaltung eines Konzepts mit hohen Anforderungen an die Einhaltung von Hygiene- und Abstandsregelungen nicht vollständig ausschließen [20].

In Anbetracht der Herausforderungen in der praktischen Implementierung von Interventionsstudien in Inklusionsbetrieben sowie einer bislang unzureichenden Studienlage ergibt sich ein weiterer Forschungsbedarf hinsichtlich zusätzlicher Studien zur BGF in Inklusionsbetrieben [22]. Nach dem aktuellen Stand der Forschung werden hierfür ganzheitliche Ansätze als am effektivsten bewertet, welche sowohl verhaltens- als auch verhältnispräventive Maßnahmen berücksichtigen [14].

### Limitationen

Während der Erhebungszeitpunkte bestanden in den kooperierenden Inklusionsbetrieben aufgrund der Pandemiesituation diverse wirtschaftliche Herausforderungen wie Personalmangel, welche die Teilnahmebereitschaft der Betriebe an der Evaluationsstudie einschränkte. Um die Aussagekraft der Evaluationsergebnisse zu erhöhen, wurde folgender Forschungsbedarf hinsichtlich des Studiendesigns identifiziert:

- Vergrößerung der Stichprobe,
- Intensivierung der Interventionen (z. B. Seminartermine in regelmäßigen Abständen),
- weitere qualitative sowie quantitative Evaluationsmethoden im Längsschnittdesign unter Einbezug von Ergebnisevaluationen, um bestehende Konzepte weiterzuentwickeln,
- Berücksichtigung der Bedürfnisse von Menschen mit unterschiedlichen Behinderungen (z. B. Evaluationsstudien mit gehörlosen Beschäftigten),
- Berücksichtigung von Branchen- und Tätigkeitsunterschieden,
- Förderung der Zusammenarbeit von kleinen und mittelständischen Inklusionsbetrieben im Rahmen von regionalen Netzwerken.

Aus dem aktuellen Stand der Forschung unterstreicht eine systematische Literaturübersicht zu Gesundheitsförderungsprogrammen für Menschen mit geistigen Behinderungen ebenso die methodischen Limitationen von Interventionsstudien hinsichtlich der Rekrutierung, der Durchführung von Interventionen, der Wahl der Ergebnisparameter und Messmethoden [31].

## Ausblick

Die vorliegende Studie liefert erste Evaluationsergebnisse zur Implementierung von verhaltenspräventiven BGF-Maßnahmen für schwerbehinderte Beschäftigte und deren Leitungskräfte in Inklusionsbetrieben. Der aktuelle Stand der Forschung verdeutlicht einen weiteren Forschungsbedarf an Interventionsstudien zur Entwicklung und Evaluation von BGF-Maßnahmen in Inklusionsbetrieben. Die Kombination von sowohl verhältnis- als auch verhaltensbezogenen Interventionen werden im Sinne einer ganzheitlichen Vorgehensweise empfohlen [31]. Unterschiedliche Studiendesigns, wie Interventions- und Langzeitstudien mit qualitativen, quantitativen oder im Mixed-Methods-Design und unter Einbezug von Ergebnisevaluationen, werden hierfür empfohlen. Die Stichproben sollten die Diversität in Inklusionsbetrieben entsprechend hinsichtlich Alter, Geschlecht, Art der Behinderung und Unternehmensgröße abbilden. Insgesamt wird im Sinne einer Partizipativen Forschung angeregt, Menschen mit Behinderungen auch in zukünftige Forschung von der Problemstellung bis zur Evaluation einzubeziehen, um ein inklusives Forschungsdesign zu gewährleisten [18].

## Fazit für die Praxis

- Die Evaluationsergebnisse zeigten auf, dass Seminare zur Förderung der psychischen Gesundheit für Beschäftigte mit Behinderungen und für Leitungskräfte eine Möglichkeit darstellen, verhaltenspräventive BGF-Maßnahmen (betriebliche Gesundheitsförderung) in Inklusionsbetrieben umzusetzen.
- Vor allem die auf die Inklusionsbetriebe angepassten Seminarinhalte konnten die Zufriedenheit der Teilnehmenden mit der BGF-Maßnahme positiv beeinflussen, sodass auch zukünftig verhaltenspräventive BGF-Maßnahmen für Inklusionsbetriebe inhaltlich sowie organisatorisch an die speziellen Bedürfnisse der Zielgruppe entwickelt oder angepasst werden sollten.
- Zukünftig besteht weiterhin Bedarf, Beschäftigte und Leitungskräfte in Inklusionsbetrieben im Rahmen von BGF-Maßnahmen für die Möglichkeiten der Gesunderhaltung und -förderung zu sensibilisieren.
- Das Bewusstsein der Geschäftsführungen über ihre gesundheitsförderliche Funktion im Betrieb ist anzuregen, da sie die Führungs- und Unternehmenskultur maßgeblich beeinflussen, eine nachhaltige Umsetzung von BGF-Maßnahmen unterstützen und Beschäftigte zu gesundheitsförderlichem Verhalten ermutigen können.
- Es wird kleinen und mittelständischen Inklusionsbetrieben empfohlen, regionale Kooperationen zu bilden, um fehlende Ressourcen auszugleichen und diese zu bündeln, sich zu vernetzen und auszutauschen.
